# LCS-Net: Learnable Color Correction and Selective Multi-Scale Fusion for Underwater Image Enhancement

**DOI:** 10.3390/s26041323

**Published:** 2026-02-18

**Authors:** Gang Li, Xiangfei Zhao

**Affiliations:** School of Computer Science and Technology, Zhejiang University of Science and Technology, Hangzhou 310023, China; 222308855020@zust.edu.cn

**Keywords:** underwater image enhancement, multi-scale perception, depthwise separable convolution, color correction

## Abstract

Underwater images are frequently degraded by wavelength-dependent absorption and scattering, which introduce strong color casts, reduce contrast, and obscure fine structures. Although learning-based enhancement methods have recently improved perceptual quality, many remain computationally intensive, limiting deployment on resource-constrained underwater platforms. To address this challenge, we propose LCS-Net, a lightweight framework for single underwater image enhancement that targets a favorable quality–efficiency trade-off. LCS-Net first applies a dynamic Learnable Color Correction Module (LCCM) that predicts image-specific correction parameters from global color statistics, enabling low-overhead cast compensation and stabilizing the input distribution. Feature extraction is conducted using efficient inverted residual blocks equipped with squeeze-and-excitation (SE) to recalibrate channel responses and facilitate detail recovery under scattering-induced degradation. At the bottleneck, a Selective Multi-Scale Dilated Block (SMSDB) aggregates complementary context via parallel dilated convolutions and global cues and adaptively reweights the fused features to handle diverse water conditions. Extensive experiments on public benchmarks demonstrate that LCS-Net achieves competitive performance, yielding a PSNR of 26.46 dB and an SSIM of 0.92 on UIEB, along with 28.71 dB and 0.86 on EUVP, while maintaining a compact model size and low computational cost, highlighting its potential for practical deployment.

## 1. Introduction

Underwater vision supports a broad range of marine applications, such as ecological monitoring, offshore infrastructure inspection, underwater archeology, and perception for autonomous underwater vehicles (AUVs). However, underwater images are often severely degraded by wavelength-dependent absorption and scattering in the water medium. In practice, long-wavelength components attenuate rapidly, while shorter wavelengths are more strongly scattered, leading to persistent blue-green color casts, haze-like veiling, reduced contrast, and loss of fine structural details [1]. These degradations, together with non-uniform illumination and particle-induced backscatter [2], can significantly impair both human interpretation and downstream vision tasks [3]. In particular, for underwater target tracking, such visual degradation introduces severe observational uncertainty, leading to trajectory drift [4] and instability under occlusion [5], thereby necessitating high-fidelity enhancement as a prerequisite for reliable robotic perception.

Underwater image enhancement (UIE) has been extensively studied to alleviate these degradations [6]. Early UIE methods mainly depended on hand-crafted priors and fusion-style enhancement, which are computationally efficient but tend to be less robust when water type, imaging depth, and illumination vary [7]. Prior-driven restoration such as red-channel recovery leverages wavelength-dependent attenuation to compensate for severe color loss and improve visibility [8]. Physics-inspired correction further refines the underwater image formation process to estimate medium effects and suppress veiling artifacts [9]. Contrast-oriented enhancement, exemplified by CLAHE, is still widely used because it can boost local contrast and partially relieve shallow-water color imbalance with minimal overhead [10].

As larger real-world benchmarks and data-driven optimization have emerged, UIE research has increasingly shifted toward deep learning, as illustrated by UTIEB, which focuses on turbid-water degradations and offers standardized evaluation data [11]. Supervised CNN-based methods benefit from paired references and architectures that integrate color correction with spatial fusion to enhance contrast and preserve fine details. When paired supervision is scarce, uncertainty-aware and unsupervised formulations have been explored to stabilize learning on unpaired data and handle ambiguous supervision in real scenes [12]. Multi-color-space modeling also proves effective for mitigating global color distortion and luminance imbalance by exploiting complementary representations such as RGB, HSV, and Lab [13]. Transformer-based designs strengthen global context modeling and are frequently combined with simplified physical priors to better address scattering-related visibility loss [14]. In parallel, progress continues through multi-domain learning strategies [15] and lightweight, frequency-aware networks that target real-time deployment on resource-limited underwater platforms [16]. Despite these advances, many models remain computationally intensive, and lightweight designs may struggle to jointly handle globally consistent color shifts and the multi-scale nature of scattering, causing residual color casts or over-smoothed structures, especially under diverse water conditions [17]. Application-oriented analyses stress that UIE should be assessed together with downstream requirements such as feature matching, reinforcing the need to balance enhancement quality and deployment efficiency.

To achieve a favorable trade-off between enhancement quality and computational efficiency, we propose LCS-Net, a lightweight network that explicitly targets global color distortion and spatially varying scattering degradations. Instead of relying on a heavy stack of dense convolutional correction layers, LCCM predicts an image-adaptive global color mapping from global color statistics, enabling low-overhead cast compensation and simplifying subsequent feature learning. Second, the backbone is constructed with efficient inverted residual learning enhanced by channel attention, which strengthens channel-wise representations and helps preserve details under non-uniform degradation. Third, at the bottleneck, we design a Selective Multi-Scale Dilated Block (SMSDB) that aggregates complementary context via parallel dilated convolutions and global cues, then adaptively reweights features to better accommodate diverse underwater conditions. Finally, a PixelShuffle-based decoder combined with residual learning reconstructs fine details while maintaining stable optimization.

The main contributions of this paper are summarized as follows:

1. To facilitate deployment on resource-constrained platforms, we propose LCS-Net, a lightweight global-to-local enhancement framework. At its core is the DF-IRB, which effectively models scattering-induced veiling and contrast attenuation with low computational overhead, achieving a favorable trade-off between quality and efficiency.
2. We develop a dynamic LCCM to efficiently rectify scene-wide chromatic distortions. By predicting image-adaptive parameters from global color statistics, LCCM provides efficient color cast correction, which stabilizes and simplifies subsequent feature learning.
3. To address the multi-scale nature of underwater artifacts, we propose an SMSDB. This module aggregates complementary context via parallel dilated convolutions and global cues, employing adaptive reweighting to emphasize the most informative scales across varying water conditions.

The remainder of the paper is organized as follows: Section 2 reviews related work on underwater enhancement and restoration. Section 3 presents the proposed LCS-Net in detail. Section 4 describes experimental settings and provides quantitative comparisons with extensive analysis. Section 5 concludes the paper and discusses limitations and future directions.

## 2. Related Works

UIE aims to compensate for wavelength-dependent absorption and scattering, which commonly lead to global color casts, reduced contrast, haze-like veiling, and texture attenuation. Existing UIE studies can be broadly organized into three lines: non-physical enhancement, physical model-based restoration, and deep learning-based enhancement. In this section, we review the literature below following representative research directions.

### 2.1. Non-Physical Image Enhancement Methods

Non-physical methods enhance underwater images through direct pixel- or color-space transformations without explicitly estimating physical parameters. Classical operations include white balance and color correction, contrast stretching, histogram-based enhancement, Retinex-inspired decomposition, sharpening, and multi-variant fusion. Such methods are typically training-free and efficient, making them convenient for practical preprocessing. A representative baseline is RGHS [18], which performs histogram and contrast-oriented processing to improve global visibility. Recent works refine global color alignment and contrast shaping via structured yet lightweight designs; for example, PCDE [19] adopts piecewise color correction with contrast optimization, while luminance decomposition and fusion pipelines reconstruct luminance from RGB components and aggregate multi-scale information to obtain balanced enhancement [20]. These designs also highlight the importance of explicit global color handling, which later inspires learning-based modules [21].

### 2.2. Physical Model-Based Restoration Methods

Physical model-based methods treat UIE as an inverse problem under an underwater image formation model, where attenuation, transmission, and backscatter jointly affect the observed image. Restoration is typically performed by estimating intermediate quantities and reconstructing the scene radiance accordingly. Representative baselines include IBLA [22], which leverages blurriness and absorption-related cues for restoration, and UDCP [23], which adapts dehazing-inspired priors to underwater scenarios for visibility recovery. Another representative framework aims to separate water effects from scene radiance and often benefits from depth or strong depth cues for reliable correction. More recent studies further revisit model assumptions and estimation strategies under complex illumination, and some incorporate auxiliary constraints to better isolate backscatter-related components [24]. Overall, physical restoration provides interpretable modeling insights and remains an important baseline in UIE evaluation.

### 2.3. Deep Learning-Based Enhancement Methods

Deep learning-based UIE methods learn enhancement mappings from data and have become prevalent due to strong representation capability. Following common practice, learning-based methods are discussed under CNN-based, GAN-based, and Transformer-based approaches [25]. We review representative designs in a coherent progression.

#### 2.3.1. CNN

CNN-based methods are widely adopted because convolutional architectures are efficient and effective for local structure recovery. Many works employ encoder–decoder backbones, multi-branch processing, or fusion-style designs to jointly improve color fidelity and visibility. In our comparisons, Water-Net [26] is a representative CNN baseline with fusion-style enhancement, and UIEC2Net [27] integrates complementary cues for improved color and structure restoration. PUIE [28] represents a prior-guided learning line that introduces additional guidance beyond plain regression. Alongside quality-oriented designs, lightweight CNNs are also actively explored: Shallow-UWnet [29] adopts a compact structure for efficient enhancement, and LiteEnhanceNet [30] targets an improved quality–efficiency trade-off. Recent compact CNNs further introduce efficient operators and training strategies, such as computation-efficient convolution with channel shuffle [31], lightweight color compensation and scalable design [32], re-parameterization for efficient inference [33], and decomposition-style pipelines that separate color and texture processing before fusion [34]. Collectively, CNN-based UIE spans from multi-branch enhancement to real-time lightweight variants.

#### 2.3.2. GAN

GAN-based UIE introduces adversarial learning to encourage visually realistic outputs and is often used when perceptual appearance is emphasized or when paired supervision is limited. FUnIE-GAN [35] is a practical baseline that emphasizes fast enhancement and is frequently adopted in underwater perception pipelines. Recent GAN variants incorporate stronger global modeling and attention mechanisms; for instance, TEGAN [36] embeds Transformer-style modules into an adversarial framework, and other efficiency-aware adversarial designs have also been explored [37]. In general, GAN-based methods complement CNN-based enhancement by providing an alternative perceptual modeling perspective.

#### 2.3.3. Transformer

Transformer-based UIE leverages attention mechanisms to capture long-range dependencies, which is beneficial for global color trends and spatially non-uniform degradations. In our comparisons, Ucolor [38] emphasizes global context modeling for underwater enhancement, and U-Shape [39] integrates Transformer-style modules into a U-shaped restoration framework to combine hierarchical reconstruction with global interactions. Recent Transformer variants further explore domain-aware feature aggregation to fuse complementary representations for underwater enhancement [40]. Overall, Transformer-based approaches offer strong global modeling priors and are increasingly combined with convolutional components to maintain practical efficiency.

In summary, non-physical methods offer simple and efficient color and contrast manipulation, physical restoration provides interpretable modeling of attenuation and scattering, and learning-based approaches improve robustness through data-driven representation learning. These developments collectively motivate lightweight designs that explicitly address global color casts and efficiently model multi-scale scattering under real-time constraints.

## 3. Proposed Approach

In this paper, we present a novel CNN-based framework, termed LCS-Net, for cost-effective enhancement of single underwater images. This section presents the architectural design and key mechanisms of the proposed method. Specifically, Section 3.1 overviews the overall network architecture of LCS-Net. Section 3.2 details the core modules, including the Learnable Color Correction Module, the feature encoder, and the Selective Multi-Scale Dilated Block. Finally, Section 3.3 describes the combined loss function used to supervise network training.

### 3.1. Network Architecture

**Figure 1:**
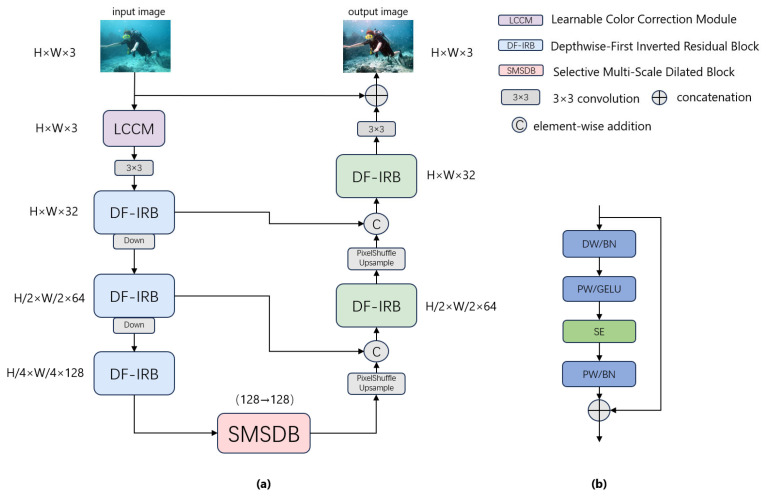
Arrows denote data flow, colors distinguish modules. (**a**) Overall architecture of the proposed LCS-Net. The network consists of an LCCM, an encoder–decoder built with DF-IRB, and an SMSDB at the bottleneck. PixelShuffle upsampling and skip connections are used for progressive reconstruction. (**b**) shows the internal design of DF-IRB.

As shown in Figure 1, LCS-Net adopts a two-stage design comprising global color correction and hierarchical feature extraction–reconstruction. First, an input-level LCCM corrects the dominant scene-wide color cast with negligible overhead, thereby simplifying subsequent representation learning. The color-corrected image is then fed into a U-shaped encoder composed of stacked DF-IRB blocks. Strided convolutions progressively downsample features to form multi-scale hierarchical representations. At the bottleneck, SMSDB aggregates complementary context via parallel dilated-convolution branches and a global context branch and then performs channel-wise recalibration to selectively fuse multi-receptive-field cues under diverse underwater degradations. The decoder uses PixelShuffle-based upsampling and concatenates the upsampled features with corresponding encoder features to progressively recover spatial details. Finally, a simple prediction head estimates a residual map, which is added to the input image to generate the enhanced output. This residual formulation stabilizes optimization while improving contrast and preserving fine structures.

### 3.2. Network Composition

#### 3.2.1. Learnable Color Correction Module (LCCM)

**Figure 2:**
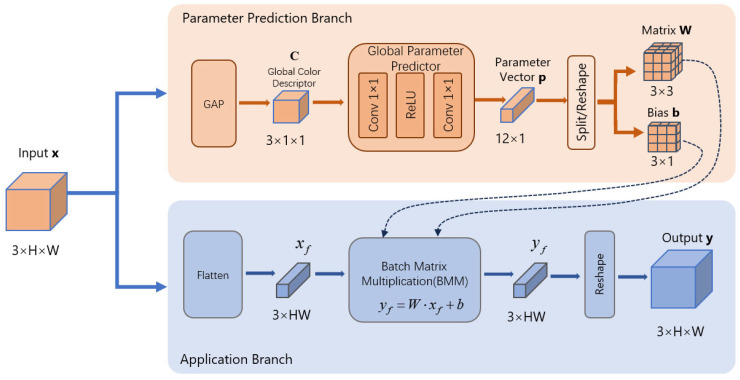
Detailed architecture of the LCCM. Arrows denote data flow, colors distinguish modules.

As shown in Figure 2, LCCM is placed at the input of LCS-Net to compensate for the dominant scene-wide color cast before deep feature extraction. Since wavelength-dependent absorption often induces a largely global chromatic shift, relying on locality-biased convolutional stacks to implicitly capture image-level color statistics is inefficient and may unnecessarily increase computation when expanding receptive fields.

LCCM formulates color rectification as an image-adaptive global affine mapping applied uniformly to all pixels. Specifically, we use global average pooling to obtain a compact global color descriptor, and a lightweight parameter predictor composed of two 1 × 1 convolutions followed by a ReLU maps it to correction parameters that define a 3 × 3 color transformation matrix and a per-channel bias. This globally shared mapping performs channel mixing and offset adjustment, producing consistent cast correction while preserving geometric structures.

This design is effective yet efficient. Conditioning on image-level statistics enables adaptation to different water types. The predicted matrix and bias provide an interpretable correction. Performing cast compensation upfront reduces the burden on the backbone, allowing it to focus on spatially varying scattering and detail restoration.

#### 3.2.2. Depthwise-First Inverted Residual Block (DF-IRB)

In turbid underwater environments, scattering induced by suspended particles often produces haze-like veiling that blurs structural boundaries and reduces contrast. To better model such spatially extended degradations under an efficiency constraint, we build upon the inverted residual paradigm and large-kernel depthwise spatial aggregation and develop a DF-IRB to strengthen contextual modeling while keeping the computation efficient. Specifically, DF-IRB starts with a 7 × 7 depthwise convolution to enlarge the effective receptive field (ERF), enabling the network to capture broader contextual cues that are beneficial for characterizing large-scale degradations such as non-uniform illumination and local turbidity. Owing to the channel-wise nature of depthwise operations, the larger kernel introduces only a modest computational overhead. After spatial aggregation, DF-IRB follows an inverted residual style channel-mixing stage: features are first expanded by a 1 × 1 pointwise convolution with an expansion ratio of r = 2, followed by a GELU nonlinearity for smoother feature transformation, and then projected back to the original channel dimension via another 1 × 1 pointwise convolution. To further emphasize informative structures and suppress irrelevant responses, we incorporate an SE module after the projection. Finally, a residual shortcut adds the block output to the input, stabilizing optimization and preserving fine details. Overall, DF-IRB serves as a compact feature extraction unit that enhances structural representation while mitigating scattering-related interference in LCS-Net.

#### 3.2.3. Selective Multi-Scale Dilated Block (SMSDB)

Underwater scattering and backscatter vary with depth, spatial location, and water type, which makes degradation inherently multi-scale. Near-field regions often preserve more textures but suffer from non-uniform illumination, while far-field regions are dominated by haze-like veiling and contrast loss. To model these effects under strict efficiency constraints [41], we introduce a Selective Multi-Scale Dilated Block, SMSDB, at the bottleneck to aggregate complementary cues across multiple effective receptive fields and to emphasize the most informative scales.

**Figure 3:**
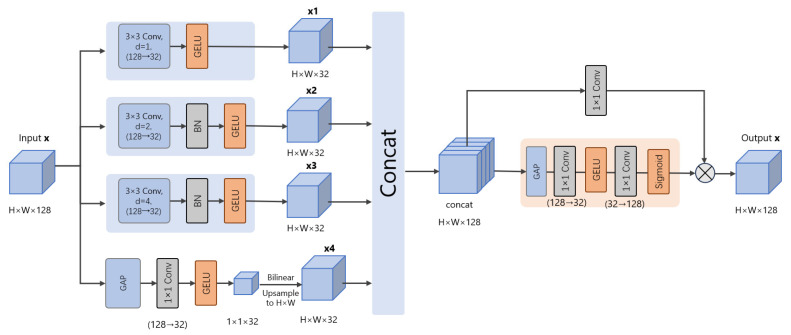
Detailed architecture of the SMSDB. Arrows denote data flow, colors distinguish modules.

As shown in Figure 3, SMSDB adopts a four-branch design. Each branch first projects the input features to a reduced-width subspace to control complexity. A standard 3 × 3 convolution captures local structures and fine textures. Two 3 × 3 dilated convolutions with dilation rates 2 and 4 capture mid-range and long-range scattering patterns without enlarging kernel size. A global context branch uses global average pooling and a 1 × 1 convolution to produce a scene-level descriptor that is expanded to the original spatial resolution, providing globally consistent guidance when degradations are coherent at the scene level.

The branch outputs are concatenated and fused by a 1 × 1 convolution to recover the target channel width and mix multi-scale features efficiently. We then apply an efficient selective channel reweighting module on the fused representation. Global pooling summarizes the fused features, and a compact gating network predicts channel-wise weights via an SE-style mapping. The fused features are reweighted accordingly, suppressing uninformative responses and amplifying the most relevant scales for each input.

Overall, SMSDB provides a compact mechanism for content-adaptive receptive field selection. By combining local texture preservation, multi-dilated context, and global scene guidance with selective reweighting, it improves robustness across diverse underwater conditions with minimal computational overhead.

### 3.3. Loss Function

To supervise LCS-Net under paired training data, we minimize a composite objective that jointly constrains pixel-wise fidelity, perceptual similarity, and structural consistency.

#### 3.3.1. Mean Squared Error (MSE) Loss

We employ the mean squared error (MSE) to enforce pixel-level fidelity between the enhanced output and the reference image:

(1) $$L_{mse}=\frac{1}{N}\sum_{x=1}^{N}\|I_{out}\left(x\right)-I_{gt}\left(x\right){\|}_{2}^{2}\;$$

where $I_{out}\left(x\right)$ and $I_{gt}\left(x\right)$ represent the pixel values of the enhanced image and the ground truth reference at position x, respectively, and N denotes the total number of pixels. This loss ensures that the low-frequency information of the image is accurately recovered.

#### 3.3.2. Structural Similarity (SSIM) Loss

To regulate the structural similarity and contrast between the generated image and the ground truth, we incorporate the SSIM loss. It is formulated as:

(2) $$\;L_{ssim}=1-\frac{1}{N}\sum_{x=1}^{N}\mathrm{SSIM}\left(I_{out}\left(x\right),I_{gt}\left(x\right)\right)$$

where SSIM compares the local patterns at the image block level based on brightness, contrast, and structure:

(3) $$\;SSIM\left(p,g\right)=\frac{\left(2\mu_{p}\mu_{g}+C_{1}\right)\left(2\sigma_{pg}+C_{2}\right)}{\left(\mu_{p}^{2}+\mu_{g}^{2}+C_{1}\right)\left(\sigma_{p}^{2}+\sigma_{g}^{2}+C_{2}\right)}$$

Here, $\mu_{p}$ and $\mu_{g}$ represent the mean intensities of patches p and g; $\sigma_{p}^{2}$ and $\sigma_{g}^{2}$ denote their variances; and $\sigma_{pg}$ is the covariance. In addition, $C_{1}={\left(255\times 0.01\right)}^{2}$ and $C_{2}={\left(255\times 0.03\right)}^{2}$ are constants that ensure numerical stability.

#### 3.3.3. Perceptual Loss

The VGG loss function is defined based on the activation of the pre-trained VGG-19 network. We feed the enhanced image and the reference image into a pretrained VGG-19 to extract feature maps, and compute an L_2_ distance between the corresponding features:

(4) $$L_{per}=\|\Phi \left(I_{out}\right)-\Phi \left(I_{gt}\right){\|}_{2}$$

where Φ represents an image feature function extracted from the specific layers of the VGG-19 network. This loss ensures consistency in high-level semantic content and textures.

Finally, the total loss function is defined as follows:

(5) $$L_{total}=L_{mse}+L_{ssim}+L_{per}$$

## 4. Experiments

This section details the experiments for LCS-Net. We first present the implementation details and benchmark settings. Next, we report quantitative and qualitative comparisons against representative prior-based and learning-based UIE methods on the UIEB and EUVP datasets. Finally, we conduct ablation studies and analyze computational complexity to validate the effectiveness of our design choices.

### 4.1. Experimental Details

Training and inference were performed on an NVIDIA GeForce RTX 4090 GPU with 24 GB VRAM using CUDA 11.8. The network was trained for 200 epochs using the Adam optimizer with a batch size of 16 and an initial learning rate of 2 × 10^−4^. A cosine-annealing schedule was adopted to decay the learning rate to 1 × 10^−6^. To increase data diversity, synchronized random rotations of 0°, 90°, 180°, and 270° were applied to each input–reference pair, which avoids interpolation-induced artifacts.

To comprehensively assess the enhancement quality and generalization ability of the proposed method, we select a set of representative baselines spanning both conventional and data-driven UIE approaches. Specifically, the conventional group includes IBLA [22], UDCP [23], and RGHS [18], covering typical physics-prior and histogram-based enhancement pipelines. The learning-based group further comprises FUnIE-GAN [35], Water-Net [26], Shallow-UWnet [29], Ucolor [38], UIEC2Net [27], PUIE [28], LiteEnhanceNet [30], and U-Shape [39], representing mainstream CNN, GAN, and Transformer paradigms. For a fair comparison, we follow the parameter settings and inference procedures reported in the original papers or released implementations as closely as possible; apart from necessary adjustments to input resolution to match the dataset protocol, all other key configurations are kept unchanged.

### 4.2. Datasets

We evaluate LCS-Net on two widely used benchmarks, UIEB [26] and EUVP [35], to validate its performance under paired supervision and diverse real-world conditions. The dataset statistics and splits are summarized in Table 1. Unless otherwise specified, all images are resized to 256 × 256 for both training and evaluation to standardize the input resolution across compared methods.

UIEB is a paired benchmark for underwater image enhancement that covers diverse degradations, including blue or green color casts, contrast attenuation, haze-like veiling caused by scattering, and non-uniform illumination. It consists of real underwater photographs collected from multiple sources such as in situ captures, online resources, and prior studies. UIEB provides human-in-the-loop references, where multiple candidates’ enhanced images are generated for each input and the visually preferred one is selected as the reference. In our experiments, we used 800 paired samples for training and the remaining 90 paired samples for testing. The 60 challenging images without references are excluded from full-reference evaluation and are used only for qualitative comparison.

EUVP is a large-scale benchmark for underwater visual perception enhancement. It provides both paired and unpaired collections with diverse scene contents, water types, and camera characteristics, comprising over 12,000 paired images and 8000 unpaired images. The paired data include three distinct subsets: Underwater Dark, which focuses on low-light imagery; Underwater ImageNet, which contains ImageNet-derived pairs generated via a CycleGAN-based distortion pipeline; and Underwater Scenes, which consists of diverse in situ underwater environments. In this work, we focus on the Underwater Scenes subset, which contains 2185 paired images. We use the entire subset without manual filtering and randomly split it into 1900 training pairs and 285 testing pairs. This subset is adopted because it is closely aligned with real-world Underwater Scenes and supports a consistent full-reference evaluation protocol commonly used in prior UIE studies. Compared with UIEB, EUVP exhibits stronger cross-device variability, serving as a complementary testbed for assessing robustness.

### 4.3. Quantitative Evaluation

#### 4.3.1. Evaluation Metrics

We evaluated enhancement quality in terms of fidelity, structural consistency, and perceptual appearance. For paired test sets, we reported MSE, PSNR, and SSIM. We also reported UIQM, a no-reference metric designed for underwater images. For cases where ground-truth targets are unavailable, we primarily relied on UIQM together with qualitative comparisons.

MSE measures the pixel-wise squared difference between the enhanced image $\hat{y}$ and the reference image y, where a lower value indicates better fidelity:

(6) $$MSE\left(\hat{y},y\right)=\frac{1}{N}\sum_{i=1}^{N}{\left({\hat{y}}_{i}-y_{i}\right)}^{2}$$

Here, N denotes the total number of pixels.

PSNR is derived from MSE and quantifies reconstruction quality in decibels:

(7) $$PSNR=10\times log_{10}\left(\frac{MAX^{2}\;}{MSE}\right)\;$$

where MAX is the maximum possible pixel value.

SSIM evaluates similarity from luminance, contrast, and structural components, and is more consistent with perceived structural preservation, as shown in Formula (3).

UIQM combines colorfulness, sharpness, and contrast components for underwater images and is defined as:

(8) $$UIQM=c_{1}\cdot UICM+c_{2}\cdot UISM+c_{3}\cdot UIConM$$

In this formulation, UICM denotes the underwater colorfulness measure, UISM denotes the sharpness measure, and UIConM denotes the contrast measure. Following Panetta et al. [42], the empirically determined weights are set to $c_{1}=0.0282$, $c_{2}=0.2953$, and $c_{3}=3.5753$, respectively.

#### 4.3.2. Results

**Figure 4:**
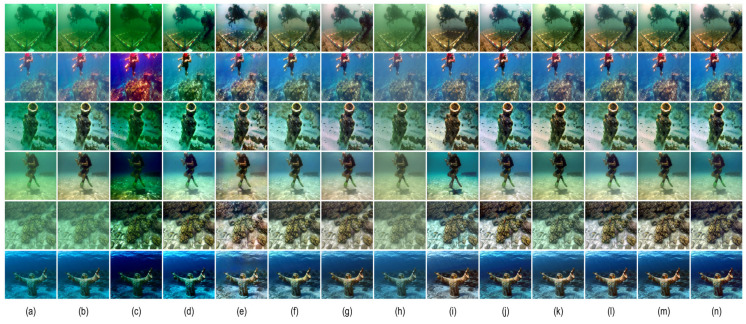
Visual comparison on the UIEB dataset. (**a**) Raw; (**b**) IBLA [22]; (**c**) UDCP [23]; (**d**) RGHS [18]; (**e**) FUnIE-GAN [35]; (**f**) Water-Net [26]; (**g**) PUIE [28]; (**h**) Shallow-UWnet [29]; (**i**) Ucolor [38]; (**j**) UIEC2Net [27]; (**k**) LiteEnhanceNet [30]; (**l**) U-Shape [39]; (**m**) Ours; (**n**) Target.

**Figure 5:**
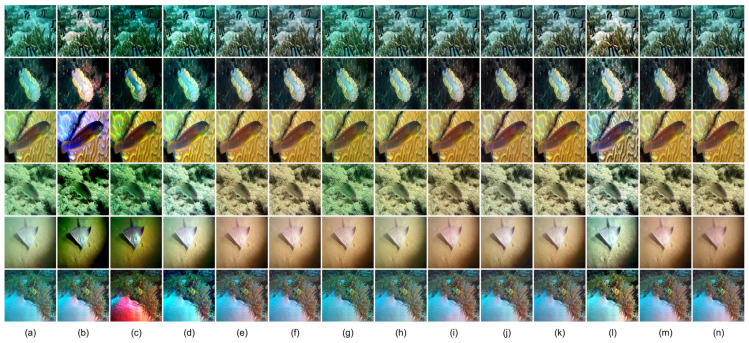
Visual comparison on the EUVP dataset. (**a**) Raw; (**b**) IBLA [22]; (**c**) UDCP [23]; (**d**) RGHS [18]; (**e**) FUnIE-GAN [35]; (**f**) Water-Net [26]; (**g**) PUIE [28]; (**h**) Shallow-UWnet [29]; (**i**) Ucolor [38]; (**j**) UIEC2Net [27]; (**k**) LiteEnhanceNet [30]; (**l**) U-Shape [39]; (**m**) Ours; (**n**) Target.

Table 2 and Table 3 report quantitative comparisons on the UIEB and EUVP benchmarks using MSE, PSNR, SSIM, and UIQM. MSE, PSNR, and SSIM are full-reference metrics that evaluate reconstruction error and structural consistency against reference images, while UIQM reflects perceptual quality in terms of color, sharpness, and contrast. On UIEB, LCS-Net achieves the best performance in MSE, PSNR, and SSIM among all compared methods, indicating accurate reconstruction and strong structural preservation under paired supervision. On EUVP, LCS-Net attains the best PSNR and SSIM, and ranks second in both MSE and UIQM, demonstrating robust generalization across more diverse scenes and imaging conditions. Overall, although LCS-Net does not always obtain the top UIQM, its perceptual quality remains highly competitive, suggesting that it enhances visibility without relying on overly aggressive saturation or contrast boosting that may introduce unnatural tones or artifacts. Representative visual results in Figure 4 and Figure 5 are consistent with these quantitative trends, and the next section provides a dedicated qualitative discussion.

To assess deployment feasibility and runtime efficiency, we benchmark representative underwater image enhancement models across varying complexity levels, ranging from lightweight GAN- and CNN-based methods to computationally intensive Transformer architectures. Table 4 presents the floating-point operations (FLOPs), parameters, and inference speeds (FPS) measured under a unified setting on an NVIDIA RTX 4090 GPU with 256 × 256 inputs. The results demonstrate that LCS-Net achieves a favorable balance between computational cost and throughput, offering significant efficiency advantages over complex baselines while maintaining competitive enhancement quality. Although specific lightweight models yield higher frame rates, they exhibit lower quantitative performance on the UIEB and EUVP datasets. Consequently, LCS-Net provides a superior trade-off between restoration fidelity and deployment efficiency.

### 4.4. Qualitative Evaluation

**Figure 6:**
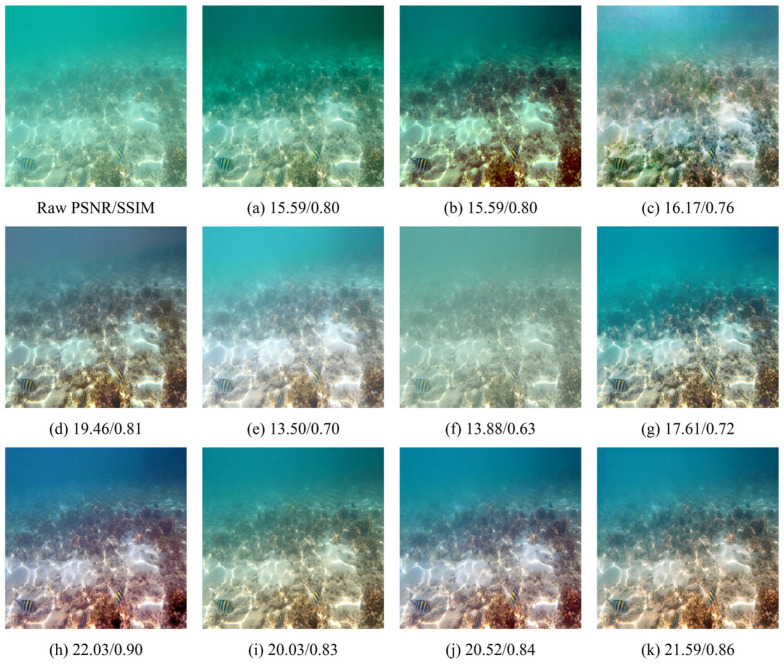
Visual comparison under heavy-scattering conditions. (**a**) IBLA [22]; (**b**) RGHS [18]; (**c**) FUnIE-GAN [35]; (**d**) Water-Net [26]; (**e**) PUIE [28]; (**f**) Shallow-UWnet [29]; (**g**) Ucolor [38]; (**h**) UIEC2Net [27]; (**i**) LiteEnhanceNet [30]; (**j**) U-Shape [39]; (**k**) Ours.

**Figure 7:**
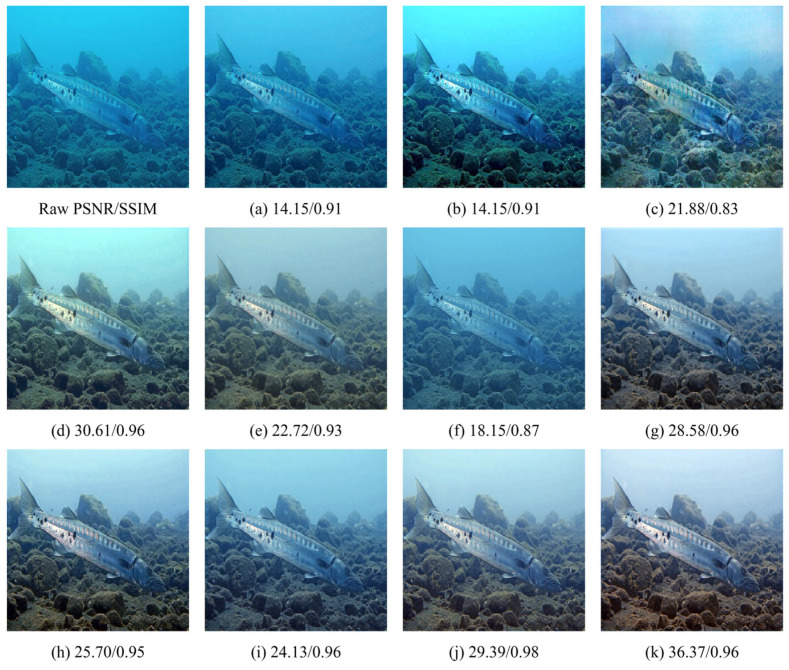
Visual comparison under strong blue-cast conditions with salient structural targets. (**a**) IBLA [22]; (**b**) RGHS [18]; (**c**) FUnIE-GAN [35]; (**d**) Water-Net [26]; (**e**) PUIE [28]; (**f**) Shallow-UWnet [29]; (**g**) Ucolor [38]; (**h**) UIEC2Net [27]; (**i**) LiteEnhanceNet [30]; (**j**) U-Shape [39]; (**k**) Ours.

**Figure 8:**
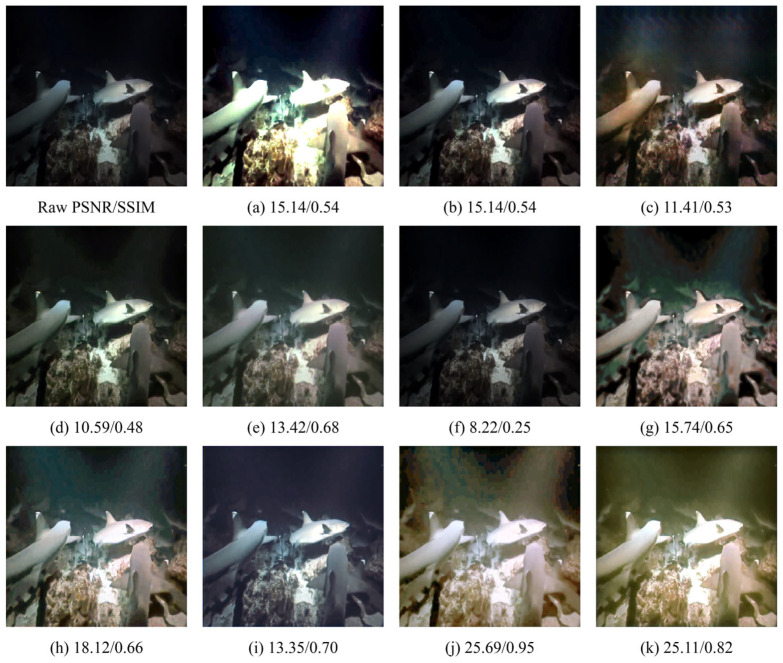
Visual comparison under low-light and non-uniform illumination conditions. (**a**) IBLA [22]; (**b**) RGHS [18]; (**c**) FUnIE-GAN [35]; (**d**) Water-Net [26]; (**e**) PUIE [28]; (**f**) Shallow-UWnet [29]; (**g**) Ucolor [38]; (**h**) UIEC2Net [27]; (**i**) LiteEnhanceNet [30]; (**j**) U-Shape [39]; (**k**) Ours.

As shown in Figure 6, Figure 7 and Figure 8, we further evaluate the visual fidelity and robustness of the proposed method under three representative and challenging underwater scenarios, including (i) heavy backscatter with low contrast and large homogeneous water-body regions (Figure 6), (ii) strong blue color cast with salient structural targets (Figure 7), and (iii) extremely degraded low-light conditions with non-uniform illumination (Figure 8). All competing methods are presented in a consistent order (Raw, (a) IBLA, (b) RGHS, (c) FUnIE-GAN, (d) Water-Net, (e) PUIE, (f) Shallow-UWnet, (g) Ucolor, (h) UIEC2Net, (i) LiteEnhanceNet, (j) U-Shape, and (k) Ours). PSNR and SSIM values are reported below each result to facilitate the interpretation of quantitative trends alongside visual observations.

In Figure 6, the degradation is mainly dominated by veiling effects caused by backscatter, leading to compressed contrast and large nearly uniform water-body regions. For such cases, an effective enhancement method should improve visibility while maintaining stable tone and luminance distribution, avoiding over-aggressive global stretching that may introduce over-whitening, tone shifts, or blocky artifacts. The results indicate that different methods make different trade-offs among dehazing strength, global tone mapping, and artifact suppression. Notably, UIEC2Net achieves higher PSNR and SSIM, reaching 22.03 and 0.90, which indicates stronger pixel-level agreement with the reference for this example. Meanwhile, our method yields slightly lower PSNR and SSIM, with values of 21.59 and 0.86, but produces a more uniform luminance distribution and a more consistent water-body tone. It also better avoids over-whitening and local block-like distortions, thereby offering a more balanced perceptual quality. In Figure 7, the scene is characterized by a dominant blue cast together with clear structural targets, which jointly evaluates color correction and structure preservation. This scenario requires the model to recover a reasonable color distribution while retaining object boundaries and textures. Compared with competing approaches, our method more effectively corrects the blue cast and enhances object–background separation, while preserving continuous textures and natural edge transitions. Consequently, it achieves the highest PSNR, reaching 36.37, together with a high SSIM of 0.96. In addition, U-Shape attains a higher SSIM of 0.98, while producing a relatively flatter contrast in this example, which suggests that a higher SSIM does not necessarily correspond to stronger perceptual sharpness or more desirable local contrast under certain conditions. In Figure 8, extremely low illumination and non-uniform lighting pose a severe challenge, where enhancement is constrained by the trade-off between visibility improvement and distortion suppression. Increasing brightness may amplify noise and induce color instability, while aggressive local enhancement can lead to saturation or loss of structural details. Different methods therefore exhibit distinct behaviors in balancing visibility improvement and artifact control. In this extreme case, U-Shape achieves the best quantitative results, reaching PSNR/SSIM of 25.69/0.95, and also produces the most favorable visual quality. By contrast, our method attains a comparable PSNR of 25.11 with a lower SSIM of 0.82, while still maintaining a stable enhancement outcome with effective artifact suppression and structure preservation compared with most competing methods. This example indicates that extremely low-light conditions remain challenging and leave room for further improvement.

Overall, the qualitative comparisons in Figure 6, Figure 7 and Figure 8 highlight that underwater image enhancement methods typically need to balance enhancement strength, color fidelity, detail preservation, and artifact suppression. Across these challenging cases, the proposed method produces more coherent color correction and contrast restoration, remains stable under strong backscatter and extreme low-light conditions, and achieves the most pronounced quantitative gains in the structural-target scenario. These results further support the effectiveness of our approach and its potential for practical deployment.

### 4.5. Ablation Study

To further demonstrate the effectiveness of each component in the proposed LCS-Net, we conducted comprehensive ablation experiments covering the network architecture, and loss functions.

#### 4.5.1. Ablation on Network Components

To validate the effectiveness of our design, we conducted a progressive ablation study on the LCCM and SMSDB modules. Integrating the LCCM into the baseline significantly boosts image fidelity and perceptual quality with negligible computational overhead, as the FLOPs remain virtually unchanged. The addition of the SMSDB further enhances structural consistency by capturing multi-scale context, albeit with a modest trade-off in inference speed. Ultimately, the full LCS-Net achieves the best overall performance by combining these complementary advantages, delivering robust restoration quality while maintaining high efficiency suitable for real-time deployment.

**Figure 9:**
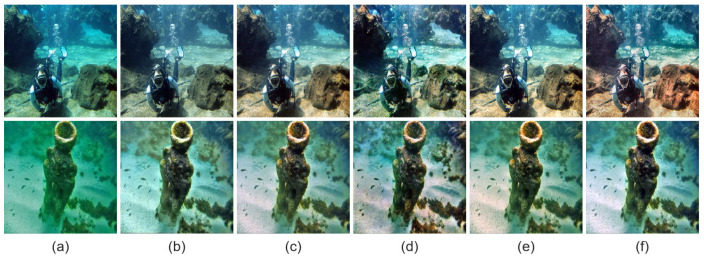
Qualitative ablation comparisons. (**a**) Input, (**b**) Base, (**c**) Base + LCCM, (**d**) Base + SMSDB, (**e**) Ours, (**f**) Reference.

As shown in Figure 9, the baseline still exhibits residual veiling haze and noticeable color artifacts. Adding LCCM markedly reduces the dominant global color cast and yields a more consistent overall tone. In contrast, adding SMSDB mainly enhances structural sharpness and local contrast, which is particularly beneficial in regions affected by scattering. Combining both modules produces clearer and more natural results, consistent with the quantitative improvements reported in Table 5. These observations further support the intended roles of LCCM and SMSDB in stabilizing global color statistics and recovering local structural details.

#### 4.5.2. Ablation on Depthwise Kernel Size

To study the effect of effective receptive field on modeling scattering-induced underwater degradations, we performed an ablation study on the kernel size of the depthwise spatial aggregation convolution in the proposed DF-IRB. Specifically, we tested kernel sizes of 3 × 3, 5 × 5, and 7 × 7 while keeping the rest of LCS-Net unchanged, including the DF-IRB configuration, the number of blocks, and all training settings. The results are reported in Table 6. This study assesses whether enlarging the depthwise kernel improves restoration quality and provides evidence supporting our final kernel-size choice.

#### 4.5.3. Ablation on Loss Functions

To quantify the relative contributions of individual loss terms, we adopt a leave-one-out ablation strategy. Using the full objective $L_{total}$ as the baseline, we remove one loss component at a time while keeping the network architecture, training protocol, and the remaining loss terms unchanged, and then evaluate the resulting model using MSE, PSNR, SSIM, and UIQM. In general, a larger performance degradation after removing a term indicates that this component plays a more critical role in driving the final enhancement quality. As shown in Table 7, removing SSIM loss term causes the most noticeable drop in SSIM and UIQM, highlighting the importance of structural consistency for preserving edge geometry, local contrast, and overall visual coherence. Removing MSE loss term leads to the largest loss in PSNR, suggesting that pixel-wise reconstruction remains the primary driver for fidelity and stable convergence. By contrast, removing perceptual loss term has a relatively limited impact on the quantitative scores, but it more often results in weaker textural details and reduced visual naturalness, indicating that perceptual supervision mainly complements high-frequency detail recovery and helps alleviate over-smoothing. Overall, jointly optimizing $L_{mse}$, $L_{ssim}$, and $L_{per}$ provides a better balance among pixel fidelity, structural preservation, and texture realism, yielding more stable and overall superior enhancement results.

## 5. Conclusions

This paper presents LCS-Net, an efficient framework for single underwater image enhancement that addresses the coupled degradations of wavelength-dependent color shift and scattering-induced contrast attenuation through a compact global–local restoration paradigm. The proposed LCCM predicts image-adaptive correction parameters from global color statistics to stabilize the input color distribution and suppress dominant casts, while the SMSDB aggregates multi-receptive-field context with selective reweighting to better model scattering haze and depth-related degradations. In addition, SE-equipped inverted residual blocks strengthen channel-wise representation and detail recovery with low computational overhead. Experiments on UIEB and EUVP demonstrate that LCS-Net achieves consistently competitive performance in PSNR, SSIM, and UIQM, delivering stable color and structure restoration while maintaining low parameter and computation budgets, which supports its suitability for deployment on resource-constrained platforms. Current limitations mainly arise in extremely low-light scenarios, where visibility enhancement may amplify sensor noise, partly due to the absence of an explicit denoising module. Future work will explore joint enhancement–denoising, unsupervised noise modeling, and semi-supervised learning with large-scale unlabeled underwater data to further improve robustness and generalization in open-water conditions.

## Figures and Tables

**Table 1:** Dataset configuration and splits.

	**UIEB**	**EUVP-Underwater_Scenes**
Training Pairs	800	1900
Testing Pairs	90	285
Image size	256 × 256	256 × 256

**Table 2:** Comparative experimental results of different methods on the UIEB dataset for enhancement performance, where the best and second-best scores are highlighted in red and blue, respectively. **↑** indicates that higher values are more desirable, and **↓** indicates that lower values are more desirable.

Methods	MSE (×10^3^) ↓	PSNR (dB) ↑	SSIM ↑	UIQM ↑
IBLA	2.64 ± 3.01	15.74 ± 5.32	0.78 ± 0.18	2.36 ± 0.25
RGHS	0.72 ± 0.88	21.01 ± 4.01	0.70 ± 0.09	2.35 ± 0.50
UDCP	1.98 ± 1.72	16.22 ± 4.80	0.69 ± 0.11	2.43 ± 0.56
FUnIE-GAN	0.68 ± 0.72	21.20 ± 2.90	0.80 ± 0.06	3.10 ± 0.34
UIEC2Net	0.47 ± 0.32	24.04 ± 4.28	0.87 ± 0.12	3.05 ± 0.42
Ucolor	0.67 ± 0.72	21.44 ± 3.63	0.88 ± 0.07	3.23 ± 0.33
WaterNet	0.56 ± 0.75	22.51 ± 3.79	0.86 ± 0.07	2.95 ± 0.42
Shallow-UWnet	1.12 ± 1.22	20.14 ± 4.73	0.81 ± 0.12	2.91 ± 0.38
PUIE	0.56 ± 0.60	22.36 ± 3.85	0.85 ± 0.07	3.21 ± 0.86
LiteEnhanceNet	0.48 ± 0.62	23.64 ± 4.87	0.89 ± 0.06	3.08 ± 0.37
U-Shape	0.46 ± 0.42	23.04 ± 3.50	0.82 ± 0.07	3.24 ± 0.35
Ours	0.34 ± 0.41	26.46 ± 4.98	0.92 ± 0.06	3.06 ± 0.33

**Table 3:** Comparative experimental results of different methods on the EUVP dataset for enhancement performance, where the best and second-best scores are highlighted in red and blue, respectively. **↑** indicates that higher values are more desirable, and **↓** indicates that lower values are more desirable.

Methods	MSE (×10^3^) ↓	PSNR (dB) ↑	SSIM ↑	UIQM ↑
IBLA	1.34 ± 1.56	19.38 ± 4.31	0.79 ± 0.19	2.36 ± 0.59
RGHS	0.74 ± 0.61	21.81 ± 3.01	0.77 ± 0.08	2. 29 ± 0.31
UDCP	1.56 ± 1.11	18.81 ± 3.53	0.67 ± 0.10	2.23 ± 0.42
FUnIE-GAN	0.32 ± 0.17	25.89 ± 2.98	0.77 ± 0.06	2.85 ± 0.49
UIEC2Net	0.60 ± 0.51	25.04 ± 4.28	0.79 ± 0.12	2.70 ± 0.40
Ucolor	0.25 ± 0.12	26.44 ± 3.73	0.81 ± 0.07	2.86 ± 0.44
WaterNet	0.76 ± 0.75	23.61 ± 3.59	0.77 ± 0.07	2.68 ± 0.48
Shallow-UWnet	0.22 ± 0.16	27.67 ± 2.98	0.82 ± 0.05	2.76 ± 0.43
PUIE	0.53 ± 0.34	21.75 ± 2.94	0.76 ± 0.05	2.74 ± 0.50
LiteEnhanceNet	0.29 ± 0.15	27.83 ± 2.92	0.83 ± 0.04	2.84 ± 0.60
U-Shape	0.35 ± 0.22	26.06 ± 3.73	0.80 ± 0.07	3.06 ± 0.48
Ours	0.24 ± 0.12	28.71 ± 2.93	0.86 ± 0.04	2.90 ± 0.40

**Table 4:** Comparison of computational complexity and inference speed with state-of-the-art methods.

Methods	FLOPs (G)	Params (M)	FPS
FUnIE-GAN	10.24	7.02	330.23
PUIE	30.09	1.41	120.11
U-Shape	26.11	31.59	33.53
Shallow-UWnet	21.65	0.22	308.32
Ours	4.10	0.45	209.60

**Table 5:** Comparison results of ablated variants with different module configurations. **↑** indicates that higher values are more desirable.

Methods	PSNR (dB) ↑	SSIM (×10^−2^) ↑	UIQM ↑	FLOPs (G)	Params (M)	FPS
Base	25.12 ± 4.81	91.83 ± 6.07	2.99 ± 0.33	3.857	0.473	240.33
Base + LCCM	25.97 ± 4.78	91.96 ± 5.92	3.05 ± 0.32	3.857	0.474	239.05
Base + SMSDB	26.02 ± 4.66	92.01 ± 5.48	3.03 ± 0.35	4.101	0.512	210.20
Ours	26.46 ± 4.98	92.29 ± 4.69	3.06 ± 0.33	4.101	0.513	209.60

**Table 6:** Comparison results of convolution kernels of different sizes. **↑** indicates that higher values are more desirable.

Kernel Size	PSNR (dB) ↑	SSIM (×10^−2^) ↑	UIQM ↑
3 × 3	26.01 ± 4.43	91.61 ± 4.53	2.99 ± 0.32
5 × 5	26.18 ± 4.71	91.81 ± 5.36	3.00 ± 0.34
7 × 7	26.46 ± 4.98	92.29 ± 4.69	3.06 ± 0.33

**Table 7:** Results of loss function ablation study. The values have the most significant decrease are highlighted in bold. The symbol "**√**" indicates that the loss term is included, and "**×**" indicates that it is excluded. **↑** indicates that higher values are more desirable.

$L_{mse}$	$L_{simm}$	$L_{per}$	PSNR (dB) ↑	SSIM (×10^−2^) ↑	UIQM ↑
**√**	**√**	**√**	26.46 ± 4.98	92.29 ± 6.69	3.06 ± 0.33
**×**	**√**	**√**	**25.95 ± 4.69**	92.13 ± 5.59	3.06 ± 0.34
**√**	**×**	**√**	25.32 ± 4.68	**91.12 ± 7.19**	3.07 ± 0.36
**√**	**√**	**×**	25.39 ± 4.69	92.24 ± 6.63	**3.04 ± 0.37**

## Data Availability

The data are from public datasets, which are introduced in Section 4.2.
